# An overview of clinical decision support systems: benefits, risks, and strategies for success

**DOI:** 10.1038/s41746-020-0221-y

**Published:** 2020-02-06

**Authors:** Reed T. Sutton, David Pincock, Daniel C. Baumgart, Daniel C. Sadowski, Richard N. Fedorak, Karen I. Kroeker

**Affiliations:** 1 Department of Medicine, Division of Gastroenterology, University of Alberta, Edmonton, Canada; 2 Chief Medical Information Office, Alberta Health Services, Edmonton, Canada

**Keywords:** Health services, Diagnosis, Drug regulation, Medical imaging

## Abstract

Computerized clinical decision support systems, or CDSS, represent a paradigm shift in healthcare today. CDSS are used to augment clinicians in their complex decision-making processes. Since their first use in the 1980s, CDSS have seen a rapid evolution. They are now commonly administered through electronic medical records and other computerized clinical workflows, which has been facilitated by increasing global adoption of electronic medical records with advanced capabilities. Despite these advances, there remain unknowns regarding the effect CDSS have on the providers who use them, patient outcomes, and costs. There have been numerous published examples in the past decade(s) of CDSS success stories, but notable setbacks have also shown us that CDSS are not without risks. In this paper, we provide a state-of-the-art overview on the use of clinical decision support systems in medicine, including the different types, current use cases with proven efficacy, common pitfalls, and potential harms. We conclude with evidence-based recommendations for minimizing risk in CDSS design, implementation, evaluation, and maintenance.

## Introduction: What is a clinical decision support system?

A clinical decision support system (CDSS) is intended to improve healthcare delivery by enhancing medical decisions with targeted clinical knowledge, patient information, and other health information.^1^ A traditional CDSS is comprised of software designed to be a direct aid to clinical-decision making, in which the characteristics of an individual patient are matched to a computerized clinical knowledge base and patient-specific assessments or recommendations are then presented to the clinician for a decision.^2^ CDSSs today are primarily used at the point-of-care, for the clinician to combine their knowledge with information or suggestions provided by the CDSS. Increasingly however, there are CDSS being developed with the capability to leverage data and observations otherwise unobtainable or uninterpretable by humans.

Computer-based CDSSs can be traced to the 1970s. At the time, they had poor system integration, were time intensive and often limited to academic pursuits.^3,4^ There were also ethical and legal issues raised around the use of computers in medicine, physician autonomy, and who would be at fault when using the recommendation of a system with imperfect ‘explainability’.^5^ Presently, CDSS often make use of web-applications or integration with electronic health records (EHR) and computerized provider order entry (CPOE) systems. They can be administered through desktop, tablet, smartphone, but also other devices such as biometric monitoring and wearable health technology. These devices may or may not produce outputs directly on the device or be linked into EHR databases.^6^

**Fig. 1:**
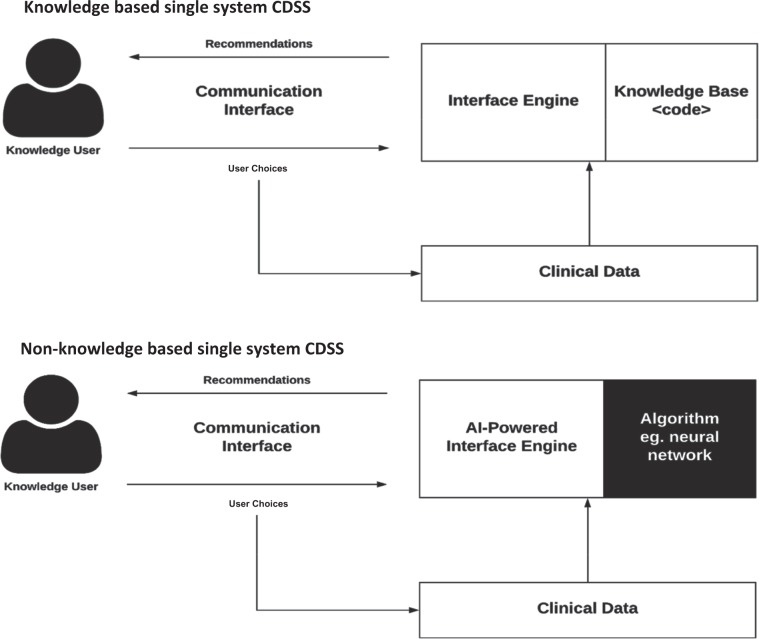
Diagram of key interactions in knowledge-based and non-knowledge based CDSS. They are composed of (1) base: the rules that are programmed into the system (knowledge-based), the algorithm used to model the decision (non-knowledge based), as well as the data available, (2) inference engine: takes the programmed or AI-determined rules, and data structures, and applies them to the patient’s clinical data to generate an output or action, which is presented to the end user (eg. physician) through the (3) communication mechanism: the website, application, or EHR frontend interface, with which the end user interacts with the system^9^.

CDSSs have been classified and subdivided into various categories and types, including intervention timing, and whether they have active or passive delivery.^7,8^ CDSS are frequently classified as knowledge-based or non-knowledge based. In knowledge-based systems, rules (IF-THEN statements) are created, with the system retrieving data to evaluate the rule, and producing an action or output^7^; Rules can be made using literature-based, practice-based, or patient-directed evidence.^2^ CDSS that are non-knowledge based still require a data source, but the decision leverages artificial intelligence (AI), machine learning (ML), or statistical pattern recognition, rather than being programmed to follow expert medical knowledge.^7^ Non-knowledge based CDSS, although a rapidly growing use case for AI in medicine, are rife with challenges including problems understanding the logic that AI uses to produce recommendations (black boxes), and problems with data availability.^9^ They have yet to reach widespread implementation. Both types of CDSS have common components with subtle differences, illustrated in Fig. 1.

CDSS have been endorsed by the US Government’s Health and Medicare acts, financially incentivizing CDS implementation into EHRs.^10^ In 2013, an estimated 41% of U.S. hospitals with an EHR, also had a CDSS, and in 2017, 40.2% of US hospitals had advanced CDS capability (HIMSS Stage 6).^11^ Elsewhere, adoption rates of EMRs have been promising, with approximately 62% of practitioners in Canada in 2013.^12^ Canada has had significant endorsement from the government level, as well as Infoway, a not-for-profit corporation.^13^ England has also been a world leader in healthcare IT investment, with up to 20 billion euros invested back in 2010.^13^ Several countries have also managed to implement national health records, at least for patient-facing data, including Denmark, Estonia, Australia, and others.^14^

The scope of functions provided by CDSS is vast, including diagnostics, alarm systems, disease management, prescription (Rx), drug control, and much more.^15^ They can manifest as computerized alerts and reminders, computerized guidelines, order sets, patient data reports, documentation templates, and clinical workflow tools.^16^ Each CDSS function will be discussed in detail throughout this review, with the potential and realized benefits of these functions, as well as unintended negative consequences, and strategies to avoid harm from CDSS. Methodology used to inform the review is shown in Box 1.

Box 1. Methods and sources used for this overview

- MEDLINE search 1980-January 2018. Key words: CDSS, diagnostic decision support system/DDSS, personal health record/PHR decision support, EHR decision support
- Hand searches of the references of retrieved literature
- University libraries searching for texts on clinical decision support systems and other keywords mentioned above
- Personal and local experience working with healthcare technology and decision support systems

## Functions and advantages of CDSS

### Patient safety

Strategies to reduce medication errors commonly make use of CDSS (Table 1). Errors involving drug-drug interactions (DDI) are cited as common and preventable, with up to 65% of inpatients being exposed to one or more potentially harmful combinations.^17^ CPOE systems are now designed with drug safety software that has safeguards for dosing, duplication of therapies, and DDI checking.^18^ The types of alerts generated by these systems are among the most disseminated kind of decision support.^19^ However, studies have found a high level of variability between how alerts for DDIs are displayed (e.g., passive or active/disruptive), which are prioritized,^20,21^ and in the algorithms used to identify DDIs.^18,22^ Systems often have varying degrees of irrelevant alerts presented, and there is no standard for how best to implement which alerts to providers. The US Office of the National Coordinator for Health Information Technology has developed a list of ‘high-priority’ list of DDIs for CDS, which has reached various levels of endorsement and deployment in CDSS’ of other countries including the U.K., Belgium, and Korea.^20,21,23^

**Table 1:** Benefits of clinical decision support systems (CDSS), possible harms, and evidence-based mitigation strategies.

1. Functions and advantages of CDSS	2A. Potential harm of CDSS	2B. Solution(s) to mitigate harm	2C. Explanation of solution(s)
**Patient Safety** Reducing incidence of medication/prescribing errors and adverse events.	**Alert fatigue** A phenomenon where too many insignificant alerts or CDSS recommendations are presented, and providers start to dismiss them regardless of importance.	Prioritize critical alerts, minimize use of disruptive alerts for non-critical indications.	Alert fatigue might be thwarted by prioritizing and selecting alerts that are critically important, that will have the greatest impact, and by tailoring alerts to specific specialties and severities (personalization).^109^ DDI testing software should ideally be programmed with an algorithm that incorporates concomitant medication, lab values, patient demographics, and administration times, to be as specific as possible.^18^
**Clinical management** Adherence to clinical guidelines, follow-up and treatment reminders, etc.	**Negative impact on user skills** One example is reliance on, or excessive trust in the accuracy of a system.	Avoid prescriptiveness in system design. Evaluate system impact on an ongoing basis.	Systems should be set up to be useful to clinicians, without jeopardizing autonomy or being too ‘prescriptive’ and definitive. It is important to conduct analysis to see how the system is being used in the long term, after implementation. If accuracy is an issue, design changes might need to be taken to prompt extra checks or confirmation of orders.^85^
**Cost containment** Reducing test and order duplication, suggesting cheaper medication or treatment options, automating tedious steps to reduce provider workload, etc.	**Financial challenges** Setup can be expensive (capital or human resource), and long-term cost-effectiveness is not guaranteed.	Design and plan for longitudinal cost analysis at the outset. Specify measurements for non-financial benefits where possible.	An analysis should be done to determine if the costs are justified and if there is a good return on investment.^110^ Cost analysis is notoriously missing in the literature, but examples can be found.^107,111,112^ Payers may be more willing to support CDSS if cost-savings can be shown elsewhere in the system / process. This means looking at more than just direct costs a using metrics such as patient outcomes or quality-adjusted life years (QALY).
**Administrative function/automation** Diagnostic code selection, automated documentation and note auto-fill.	**System and content maintenance challenges** As practice changes, there can be difficulty keeping the content and knowledge rules that power CDSS up to date.	(1) Knowledge Management (KM) Service in place, with a focus on translation to CDSS systems. (2) System for measurement and analysis of CDSS performance.	(1) Facilitates scheduled review, methods for acquiring and implementing new knowledge, and streamlined processes for gathering physician feedback on the system as well as training users on why certain data entry and standardization of data entry practices. Standards for organizing KM management have been published.^113,114^ (2) It is important to identify changes in performance and use over time. In addition, the quality of the data repository should be monitored and it is also important to ensure that conclusions are not being made on corrupted or poor quality data beforehand.^115^
**Diagnostics support** Providing diagnostic suggestions based on patient data, automating output from test results.	**User distrust of CDSS** Users may not agree with the guideline provided by the CDSS.	Reference expert knowledge—include scientific references in messages where appropriate.	To provide a verifiable source of information to the user on why the recommendation exists.^116^ In addition to increasing trust, this may provide direction for users to update their knowledge in case they were not aware of the recommendation. Many systems also query reasons for not following a recommendation in order to elucidate the source of mistrust.^117^ This is a good idea, but should not be mandatory or ‘bulky’ in design.
**Diagnostics Support: Imaging, Laboratory, and Pathology** Augmenting the extraction, visualization, and interpretation of medical images and laboratory test results.	**Transportability/interoperability** CDSS face challenges regarding integration with other hospitals or systems, making it inefficient for otherwise high-quality systems to be disseminated and scaled.	(1) Adoption of industry standards. (2) Secure cloud services and blockchain.	Major open standards for structural and semantic interoperability and exchange continue to be developed and improved by organizations such as Health Level 7 International (HL7),^118^ SNOMED International,^119^ Digital Imaging and Communications (DICOM) for imaging standards, and many others.^120^ As much as possible, these standards should be adopted at all levels within the healthcare organization, and with the external systems being used. Cloud-based EHR architecture allows for more open architecture, and flexible connectivity between systems. As with any medical system, security must be assured through compliance with legislation such as Health Insurance Portability and Accountability Act (HIPAA) in the USA, Personal Information Protection and Electronic Documents Act (PIPEDA) in Canada, and the Data Protection Directive and General Data Protection Regulation (GDPR) in Europe. In the future, we may also see blockchain used to enable greater interoperability and improve security for health information exchange (HIE).^121,122^
**Patient decision support** Decision support administered directly to patients through personal health records (PHR) and other systems.	**Dependency on computer literacy** CDSS may require a very high technological proficiency to use	(1) Conform to existing functionality. (2) Adequate training made available at launch.	(1) Maintaining consistency with the user interface of the pre-existing system (if there is one) is crucial to ensure users don’t have a steep learning curve to use the system. (2) Adequate training should be available and easily accessible for users. Training should ideally be done in person by a clinician leader with vast EHR experience to generate buy-in.^123^ Training needs to be available on an ongoing basis, as new staff and users join. One strategy is to have on-site team members designated as elite users, and capable of providing training sessions.
**Better Documentation**	**Inaccurate and poor-quality data/documentation** CDSS may aggregate data from multiple sources that are not synced properly. Users may develop manual workarounds that compromise data.	(1) Expert Knowledge of interlinked systems. (2) IT testing/debugging during development and implementation stage.	The team needs to be familiar and have expert knowledge of all external systems that feed data into the database used by the CDSS. Experts recommend testing clinical rules for PPV and NPV during the process of development and implementation.^109^ If user generated data is an issue, it may be that physicians have not received the proper training on how to read, interpret and respond to alerts, or are depending on pharmacists to check medication orders before dispensation.^124,125^
**Workflow improvement** CDSS can improve and expedite an existing clinical workflow in an EHR with better retrieval and presentation of data.	**Disrupted/fragmented workflow** CDSS can also disrupt existing workflows if they require interaction external to the EHR, or don’t match the providers’ real world information processing sequences.	(1) Usability evaluation. (2) Workflow modeling.	(1) Rigorous and iterative usability evaluations and pilot testing should be conducted on CDSS before using them in clinical settings. Many usability assessment tools are available, along with other quantitative methods and frameworks.^126–129^ (2) Unless a goal of the CDSS is to change the care process, the CDSS should be designed to fit within or conform to the current user workflows.

Other systems targeting patient safety include electronic drug dispensing systems (EDDS), and bar-code point-of-care (BPOC) medication administration systems.^24^ These are often implemented together to create a ‘closed loop’, where each step of the process (prescribing, transcribing, dispensing, administering) is computerized and occurs within a connected system. At administration, the medication is automatically identified through radio-frequency identification (RFID) or barcodes and crosschecked with patient information and prescriptions. This presents another target for CDSS and the potential benefit is the prevention of medication administration errors occurring at the ‘bedside’ (opposed to further upstream). Adoption is relatively low, partly due to high technology requirements and costs.^25^ However; studies show good efficacy for these systems in reducing errors.^26^ Mohoney et al. showed that many of these systems can be combined with CPOE and CDSS simultaneously, with reduced prescribing error rates for drug allergy detection, excessive dosing, and incomplete or unclear ordering.^24^ As with most CDSS, errors can still be made if providers omit or deliberately work around the technology.^27^

CDSS also improve patient safety through reminder systems for other medical events, and not just those that are medication related. Among numerous examples, a CDSS for blood glucose measurement in the ICU was able to decrease the number of hypoglycemia events.^28^ This CDSS automatically prompted nurses to take a glucose measurement according to a local glucose monitoring protocol, which specified how often measurements should be done according to specific patient demographics and previous glucose levels/trends.^28^

Overall, CDSS targeting patient safety through CPOE and other systems have generally been successful in reducing prescribing and dosing errors, contraindications through automated warnings, drug-event monitoring and more.^29^ Patient safety can be considered a secondary objective (or requirement) of almost all types of CDSS, no matter the primary purpose for their implementation.

### Clinical management

Studies have shown CDSS can increase adherence to clinical guidelines.^30^ This is significant because traditional clinical guidelines and care pathways have been shown to be difficult to implement in practice with low clinician adherance.^31,32^ The assumption that practitioners will read, internalize, and implement new guidelines has not held true.^33^ However, the rules implicitly encoded in guidelines can be literally encoded into CDSS. Such CDSS can take a variety of forms, from standardized order sets for a targeted case, alerts to a specific protocol for the patients it pertains to, reminders for testing, etc. Furthermore, CDSS can assist with managing patients on research/treatment protocols,^34^ tracking and placing orders, follow-up for referrals, as well as ensuring preventative care.^35^

CDSS can also alert clinicians to reach out to patients who have not followed management plans, or are due for follow-up, and help identify patients eligible for research based on specific criteria.^36^ A CDSS designed and implemented at Cleveland Clinic provides a point-of-care alert to physicians when a patient’s record matches clinical trial criteria.^37^ The alert prompts the user to complete a form which establishes eligibility and consent-to-contact, forwards the patient’s chart to the study coordinator, and prints a clinical trial patient information sheet.

### Cost containment

CDSS can be cost-effective for health systems, through clinical interventions,^38^ decreasing inpatient length-of-stay, CPOE-integrated systems suggesting cheaper medication alternatives,^39^ or reducing test duplication. A CPOE-rule was implemented in a pediatric cardiovascular intensive care unit (ICU) that limited the scheduling of blood count, chemistry and coagulation panels to a 24-h interval.^40^ This reduced laboratory resource utilization with a projected cost savings of $717,538 per year, without increasing length of stay (LOS), or mortality.

CDSS can notify the user of cheaper alternatives to drugs, or conditions that insurance companies will cover. In Germany, many inpatients are switched to drugs on hospital drug formularies. After finding that 1 in 5 substitutions were incorrect, Heidelberg hospital developed a drug-switch algorithm and integrated it into their existing CPOE system.^41^ The CDSS could switch 91.6% of 202 medication consultations automatically, with no errors, increasing safety, reducing workload and reducing cost for providers.

### Administrative functions

CDSS provide support for clinical and diagnostic coding, ordering of procedures and tests, and patient triage. Designed algorithms can suggest a refined list of diagnostics codes to aid physicians in selecting the most suitable one(s). A CDSS was conceived to address inaccuracy of ICD-9 emergency department(ED) admission coding (ICD is International Statistical Classification of Diseases, standardized codes used to represent diseases and diagnoses).^42^ The tool used an anatomographical interface (visual, interactive representation of the human body) linked to ICD codes to help ED physicians accurately find diagnostic admission codes faster.

CDSS can directly improve quality of clinical documentation. An obstetric CDSS featured an enhanced prompting system, significantly improving documentation of indications for labor induction and estimated fetal weight, compared to control hospital.^43^ Documentation accuracy is important because it can directly aid clinical protocols. For example, a CDSS was implemented to ensure patients were properly vaccinated following splenectomy, to combat the increased risk of infections (including pneumococcal, *Haemophilus influenzae*, meningococcal, etc.) that comes with spleen removal. However, the authors found that 71% of patients with the term ‘splenectomy’ in their EHR did not have it documented on their problem list (which was what triggers the CDSS alert).^44^ A supplemental CDSS was then developed to enhance problem list documentation of splenectomy,^45^ and improve the utility of the original vaccination CDSS.

### Diagnostics support

CDSS for clinical diagnosis are known as diagnostic decision support systems (DDSS). These systems have traditionally provided a computerized ‘consultation’ or filtering step, whereby they might be provided data/user selections, and then output a list of possible or probable diagnoses.^46^ Unfortunately, DDSS have not had as much influence as other types of CDSS (yet) for reasons including negative physician perceptions and biases, poor accuracy (often due to gaps in data availability), and poor system integration requiring manual data entry.^47,48^ The latter is improving with better EHR-integration and standardized vocabulary like Snomed Clinical Terms.

A good example of an effective DDSS is one which was created by Kunhimangalam et al.^49^ for diagnosis of peripheral neuropathy using fuzzy logic. Through 24 input fields which include symptoms and diagnostic test outputs, they achieved 93% accuracy compared to experts at identifying motor, sensory, mixed neuropathies, or normal cases. While this has great utility, especially in countries with less access to established clinical experts, there is also a desire for systems that can supplement specialist diagnostics. DXplain is an electronic reference based DDSS that provides probable diagnosis based on clinical manifestations.^50^ In a randomized control trial involving 87 family medicine residents, those randomized to use the system showed significantly higher accuracy (84% vs. 74%) on a validated diagnosis test involving 30 clinical cases.^50^

Given the known incidence of diagnostic errors, particularly in primary care,^51^ there is a lot of hope for CDSS and IT solutions to bring improvements to diagnosis.^52^ We are now seeing diagnostic systems being developed with non-knowledge-based techniques like machine learning, which may pave the way for more accurate diagnosis. The Babylon AI powered Triage and Diagnostic System in the U.K. is a good example of the potential, but also of the work that still has to be done before these systems are ready for primetime.^53,54^

#### Diagnostics support: imaging

Knowledge-based imaging CDSS are typically used for image ordering, where CDSS can aid radiologists in selecting the most appropriate test to run, providing reminders of best practice guidelines, or alerting contraindications to contrast, for example.^55^ An interventional CDS for image ordering at Virginia Mason Medical Center was shown to substantially decrease the utilization rate of lumbar MRI for low back pain, head MRI for headache, and sinus CT for sinusitis.^56^ The CDS required a series of questions to be answered by providers prior to image ordering (POC), to verify appropriateness. Importantly, if an image was denied, an alternative was suggested by the system. Another commercialized example is RadWise®, which guides clinicians to the most relevant imaging order by analyzing patient symptoms and matching them with a large database of diagnoses, while also providing appropriate use recommendations at the point of care.^57^

There is great interest in non-knowledge based CDS for enhanced imaging and precision radiology (‘radiomics’).^58,59^ With images accounting for increasing amounts of medical data, but requiring extensive manual interpretation, providers need technologies to aid them in extracting, visualizing, and interpreting.^60^ AI technologies are proving capable of providing insights into data beyond what humans can.^61^ To do so, these technologies make use of advanced pixel recognition and image classification algorithms, most prominently: deep learning (DL).^62^ IBM Watson Health, DeepMind, Google, and other companies are at the forefront, developing products for use in tumor detection,^63^ medical imaging interpretation,^64^ diabetic retinopathy diagnosis,^65^ Alzheimer’s diagnosis through multimodal feature learning,^62^ and countless more. IBM Watson’s ‘Eyes of Watson’, has been able to combine image recognition of a brain scan with text recognition of case descriptions to provide comprehensive decision support (or what IBM describes as a ‘cognitive assistant’).^60^

Several projects have been able to demonstrate performance that is disputably ‘on par’ with human experts.^65–68^ For example, Google’s team trained a deep convolutional neural network (CNN) to detect diabetic retinopathy (blood vessel damage in the eye) from a dataset of 130,000 retinal images with a very high sensitivity and specificity.^65^ The algorithms performance was on par with US board certified ophthalmologists. Another study just recently published by the Stanford group demonstrated a CNN for detecting arrhythmias on electrocardiogram that exceeded the accuracy (F1 and sensitivity with matched specificity) of the average cardiologist on all rhythm classes.^68^ With the current rate of progress, some experts controversially speculate that in 15–20 years, the majority of diagnostic imaging interpretation will be done (or at least pre-processed) by computers.^69^ For the time being however, we should think of these early systems as an addition or augmentation to a clinician’s available toolset.

#### Diagnostics support: laboratory and pathology

Another subset of diagnostics where CDSS can be useful is laboratory testing and interpretation. Alerts and reminders for abnormal lab results are simple and ubiquitous in EHR systems. CDSS can also extend the utility of lab-based tests for the purpose of avoiding riskier or more invasive diagnostics. In Hepatitis B and C testing, liver biopsies are considered the gold standard for diagnosis, while non-invasive lab tests are not accurate enough to be accepted. However; AI models are being developed that combine multiple tests (serum markers, imaging, and gene tests) to produce much greater accuracy.^70^ There is also application for CDSS as an interpretation tool where a test’s reference ranges are highly personalized, for example age, sex, or disease subtypes.^71^

Pathology reports are crucial as decision points for many other medical specialties. Some CDSS can be used for automated tumor grading. This was done for urinary bladder tumor grading and estimating recurrence, with up to 93% accuracy.^72^ The same has been done for brain tumor classification and grading.^73^ There are many other examples including computerized ECG analysis, automated arterial blood gas interpretation, protein electrophoresis reports, and CDSS for blood cell counting.^46^

### Patient-facing decision support

With the advent of the ‘Personal Health Record’ (PHR), we are seeing CDS functionality integrated, similar to EHRs, with the patient as the end user or ‘manager’ of the data. This is a great step towards patient-focused care, and CDS-supported PHRs are the ideal tool to implement shared decision-making between patient and provider, specifically because CDSS can remove a ‘lack of information’ as a barrier to a patient’s participation in their own care.^74^ PHRs are frequently designed as an extension of commercial EHR software, or as standalone web-based or mobile-based applications.^75^ When connected to EHRs, PHRs can have a two way relationship, whereby information entered directly by the patient can be available to their providers, and also information in the EHR can be transmitted to the PHR for patients to view.^76^

One of the earliest PHRs, the “Patient Gateway”, was simply a dashboard for patients to view medications and labs, and communicate with their physicians.^77^ This has expanded and some systems now allow patients to modify their own record of care, effecting the EHR data as well.^78^ Another example is Vanderbilt University’s MyHealthAtVanderbilt, a PHR fully integrated into the institutional EHR. In addition to disease-targeted delivery of patient educational materials, they incorporated a Flu Tool for patients with flu-like symptoms to decide the level of care they need and then help them seek treatment.^79^ Symptom tracking is a useful and common feature of PHRs, but the variety of collected data is virtually limitless, from allergies to insurance coverage to prescription and medication information.^80^ Furthermore, PHRs and other patient monitoring applications can be designed to collect information from health devices and other wearables, to create actionable insights for providers. An excellent example exists in diabetes care. Many systems are already in use,^81^ but one in particular pioneered by the Stanford School of Medicine uses a wearable glucose monitor which transmits data to an Apple device (HealthKit).^82^ Apple has made HealthKit interoperable with the Epic EHR and Epic PHR, “MyChart”. This successfully allows providers to monitor glucose trends in their patients in between visits, and contact them through MyChart for follow up or urgent recommendations. The pilot study demonstrated improved provider workflow, communication with patients, and ultimately quality of care.^82^ Various other medical fields are deploying similar systems for monitoring that combines PHR/EHR, wearable technologies, and CDSS, including but not limited to heart failure (cardiology), hypertension, sleep apnea, palliative/elder care, and more.

It is worth noting that as PHRs have become more advanced with CDSS capabilities, there has also been increasing emphasis on the design of these systems to serve shared decision making between patient and provider, and to be interactive tools to make patients more knowledgeable/involved in their own care. PHRs that only serve as a repository for health information are now seen as missing the mark, particularly by patients themselves.^75^

## Pitfalls of CDSS

### Fragmented workflows

CDSS can disrupt clinician workflow, especially in the case of stand-alone systems. Many early CDSS were designed as systems that required the provider to document or source information outside their typical workspace. CDSS also disrupt workflow if designed without human information processing and behaviors in mind. In response, CDSS have been designed using the ‘think-aloud’ method to model practitioners’ workflow and create a system with better usability.^83^

Disrupted workflow can lead to increased cognitive effort, more time required to complete tasks, and less time face-to-face with patients. Even when CDSS are well integrated within existing information systems, there can be disconnect between face-to-face interactions and interaction with a computer workstation. Studies have found that practitioners with more experiential knowledge are less likely to use, and more likely to override CDSS.^84^

### Alert fatigue and inappropriate alerts

Studies have found up to 95% of CDSS alerts are inconsequential, and often times physicians disagree with or distrust alerts.^85^ Other times they just do not read them. If physicians are presented with excessive/unimportant alerts, they can suffer from alert fatigue.^86^

Disruptive alerts should be limited to more life-threatening or consequential contraindications, such as serious allergies. However; even allergy alerts can be incorrect, and clinicians will often verify themselves, especially if the source is another site/hospital/practitioner.^85,87^ Medication alerts can also be specialty specific, but irrelevant when taken out of context. For example, an alert against using broad-spectrum antibiotics such as vancomycin may be inappropriate in ICU.^85^ An alert against duplicate medications may be inappropriate in inflammatory bowel disease clinics, where the same class of drug can be applied through different administration routes for increased effect.

### Impact on user skill

Prior to CPOE and CDSS, healthcare providers, pharmacists, and nurses were relied upon exclusively to double-check orders. CDSS can create the impression that verifying the accuracy of an order is unnecessary or automatic.^85^ This is an important myth to dispel.

It is also important to consider the potential long-term effect of a CDSS on users. Over time a CDSS can exert a training effect, so that the CDSS itself may no longer be required. Coined the “carry-over effect”, it is most likely with CDSS that are educational in nature.^88^ Conversely, providers may develop too much reliance or trust on a CDSS for a specific task.^89^ This could be compared to using a calculator for mathematical operations over a long period of time, and then having poorer mental math skills. It is potentially problematic as the user has less independence and will be less equipped for that task should they switch to an environment without the CDSS.

### CDSS may be dependent on computer literacy

Lack of technological proficiency can be hindering when engaging with a CDSS.^90,91^ This can vary by the design details of the CDSS, but some have been found to be overly complex, relying too much on user skill.^90,92^ Systems should aim to stay as close to the core functionality of the pre-existing system as possible. Regardless, all new systems have a learning period, and so baseline evaluations of users’ technological competence may be appropriate. Further training can then be provided to facilitate full use of CDSS capabilities,^93^ or more explicit guidance incorporated into the CDSS’ recommendations themselves.^94^ This information could be implemented as info buttons to be non-disruptive.^95^

### System and content maintenance

Maintenance of CDSS is an important but often neglected part of the CDSS life-cycle. This includes technical maintenance of systems, applications and databases that power the CDSS. Another challenge is the maintenance of knowledge-base and its rules, which must keep apace with the fast-changing nature of medical practice and clinical guidelines. Even the most advanced healthcare institutions report difficulty keeping their systems up to date as knowledge inevitably changes.^85^ Order sets and the algorithmic rules behind the CDSS have been identified as particularly difficult.^85^

### Operational impact of poor data quality and incorrect content

EHRs and CDSSs rely on data from external, dynamic systems and this can create novel deficiencies. As an example, some CDSS modules might encourage ordering even when the hospital lacks adequate supplies. In a study by Ash et al.^85^, a number of experts indicated that at their hospital, Hemoccult tests or pneumococcal vaccine inventories run out quickly, but this is not communicated to the CDSS.

Medication and problem lists can be problematic, if not updated or used appropriately. At one site, the medication list might be a list of dispensations, which means patients may or may not be taking them(and thus must still be asked in person).^85^ Other medication lists are generated from CPOE orders only, thus still requiring manual confirmation that patients are taking the medication. Systems that make it easy to distinguish these are ideal. It is also a major area where PHRs could create a solution, by collecting medication adherence data directly from patients.

In poorly designed systems, users may develop workarounds that compromise data, such as entering generic or incorrect data.^85^ The knowledge base of CDSS is dependent on a centralized, large clinical data repository. Quality of data can affect quality of decision support. If data collection or input into the system is unstandardized, the data is effectively corrupted. You may design a system for use at the point-of-care, but when applied to real world environments and data, will not be utilized properly. The importance of using informational standards such as ICD, SNOMED, and others, cannot be understated.

### Lack of transportability and interoperability

Despite ongoing development for the better part of three decades, CDSS (and even EHRs in general) suffer from interoperability issues. Many CDSS exist as cumbersome stand-alone systems, or exist in a system that cannot communicate effectively with other systems.

What makes transportability so difficult to achieve? Beyond programming complexities that can make integration difficult, the diversity of clinical data sources is a challenge.^96^ There is a reluctance or perceived risk associated with transporting sensitive patient information. Positively, interoperability standards are continuously being developed and improved, such as Health Level 7 (HL7) and Fast Healthcare Interoperability Resources (FHIR). These are already being utilized in commercial EHR vendors.^97^ Several government agencies, medical organizations and informatics bodies are actively supporting and some even mandating the use of these interoperability standards in health systems.^98–100^

The cloud also offers a potential solution to interoperability (and other EHR ailments such as data sync, software updating, etc.^101^). Cloud EHRs have open architecture, newer standards, and more flexible connectivity to other systems.^102^ It is also a common misconception that data stored on a cloud is more vulnerable. This is not necessarily true. Web-based EHRs are required to store data in high-level storage centers with advanced encryption and other safeguards. They must comply with national data security standards including the Health Insurance Portability and Accountability Act (HIPAA) in the USA, Personal Information Protection and Electronic Documents Act (PIPEDA) in Canada, or the Data Protection Directive and General Data Protection Regulation (GDPR) in Europe, to name a few.^103^ They can be just as safe (or just as vulnerable) as traditional, server-based architecture.^103^ In fact, there are often fewer people who have access to unencrypted data in cloud storage centers vs. server-based records.^103^

### Financial challenges

Up to 74% of those with a CDSS said that financial viability remains a struggle.^104^ Outset costs to set up and integrate new systems can be substantial. Ongoing costs can continue to be an issue indefinitely as new staff need to be trained to use the system, and system updates are required to keep pace with current knowledge.

Results from cost analyses of CDSS implementations are mixed, controversial, and sparse.^105–108^ Whether an intervention is cost-effective depends on a wide range of factors, including those specific to the environment, both political and technological.^105^ Cost benefit assessment in itself can be limited, with challenges such as a lack of standardized metrics.^107^ This is an emerging research area and much work needs to be done to advance our understanding of the financial effects of CDSS.

## Conclusion

CDSS have been shown to augment healthcare providers in a variety of decisions and patient care tasks, and today they actively and ubiquitously support delivery of quality care. Some applications of CDSS have more evidence behind them, especially those based on CPOE. Support for CDSS continues to mount in the age of the electronic medical record, and there are still more advances to be made including interoperability, speed and ease of deployment, and affordability. At the same time, we must stay vigilant for potential downfalls of CDSS, which range from simply not working and wasting resources, to fatiguing providers and compromising quality of patient care. Extra precautions and conscientious design must be taken when building, implementing, and maintaining CDSS. A portion of these considerations were covered in this review, but further review will be required in practice, especially as CDSS continue to evolve in complexity through advances in AI, interoperability, and new sources of data.
